# Intratympanic application of triamcinolone in sudden hearing loss—radiologic anatomy in cone beam CT and its’ correlation to clinical outcome

**DOI:** 10.1007/s00405-020-05920-0

**Published:** 2020-03-23

**Authors:** Willi Roßberg, Friedrich Goetz, Max Eike Timm, Thomas Lenarz, Victor Helmstaedter

**Affiliations:** 1 Department of Otorhinolaryngology, Hannover Medical University, Carl-Neuberg-Str. 1, 30625 Hannover, Germany; 2 Department of Neuroradiology, Hannover Medical University, Carl-Neuberg-Str. 1, 30625 Hannover, Germany

**Keywords:** Sensorineural hearing loss, Cone-beam CT, Triamcinolone, Intratympanic injection

## Abstract

**Purpose:**

To evaluate temporal bone cone-beam CT in patients with idiopathic sudden sensorineural hearing loss (ISSNHL) being treated with primary and secondary intratympanic (IT) triamcinolone and to possibly correlate these results to the clinical outcome.

**Methods:**

Retrospective analysis of patients treated with IT triamcinolone for ISSNHL at our department in 2018. Pre- and post-therapeutic audiologic examinations included four-tone average (FTA) at 0.5, 1, 2 and 3 kHz. Using a clinical questionnaire, pre-therapeutic CBCT scans were re-evaluated looking at items, which might interfere with adequate drug diffusion into the inner ear (e.g. bony overhangs or secondary membranes at the round or oval window).

**Results:**

Thirty-one patients were included. Twenty-four (77%; group A) had experienced ineffective systemic steroid therapy before and seven (23%; group B) received primary IT injections. Four group A-patients (21%) and two group B-patients (33%) showed a post-therapeutic FTA improvement of more than 15 dB HL. Bony overhangs at the round window niche (RWN) were present in seven cases (26%), a secondary membrane at the RWN in four (15%) and soft tissue in eight (30%) cases, respectively.

**Conclusion:**

Most patients present radiological findings in CBCT imaging, which might interfere with drug diffusion through the RW membrane. Interestingly, soft or bony tissue obstructing the RWN or the OWN was found in 50% of patients, who showed improvement of hearing. We conclude that radiologic ‘tiny’ findings are either clinically irrelevant or improvement in hearing is independent from intratympanic drug delivery.

## Introduction

Intratympanic (IT) application of glucocorticosteroids for the treatment of idiopathic sudden sensorineural hearing loss (ISSNHL) was introduced into clinical routine not only as a secondary treatment option after failure of systemic steroid treatment but also as a primary therapy in patients with contraindications to systemic treatment [1–7]. Motivation is found in blinded, placebo-controlled studies, which show significant benefits of IT steroids in comparison to intratympanic normal saline injections [8, 9] and in multiple other randomized and non-randomized studies. However, published protocols differ in the kind of steroid used, drug concentration and applied volume, number of injections and intervals between injections, total duration of treatment and starting time after the onset of ISSNHL. It becomes difficult to compare the results and subsequently to find clear recommendations for primary or secondary IT steroid treatment [5, 6, 10, 11].

Regarding the German S1-guideline ‘Treatment of acute idiopathic SSNHL’ the extent of hearing loss is not clearly defined in decibel. The subjective report of the patient in combination with the results of pure tone audiometry (PTA) is appreciated with the importance of higher relevance [12]. Others diagnose SSNHL as a decrease in the hearing of at least 30 decibel (dB) affecting at least three consecutive frequencies [13, 14].

It is generally accepted, that IT steroid injection maximizes intracochlear steroid concentration via the round window membrane (RWM) [15]. The application of higher doses of glucocorticoids will consequently lead to higher concentrations in the perilymph [16–18]. However, there are several factors that could influence the effective drug concentration and drug contact time at the round window membrane as the mean proposed transport route to the inner ear. These, for instance, include false membranes at the RW, bony overhangs and soft tissue masses. Rapid clearance of intratympanic glucocorticoid from the middle ear occurs via the Eustachian tube. In addition, drugs might be captured and eliminated by the middle ear mucosa [6, 19, 20].

Therefore, it is reasonable to check the individual anatomy through preoperative imaging of the temporal bone including cone-beam computed tomography (CBCT) and magnetic resonance imaging (MRI). To rule out anatomic variations like high riding jugular vein bulbs and other pathologies like vestibular schwannomas, we routinely perform temporal bone CBCT and MRI before IT steroid application. To our knowledge, general recommendations on pre-therapeutic radiologic imaging do not exist.

During planning IT treatment, we repeatedly identified middle ear findings like bony overhangs or soft tissue overgrowth at the round window niche (RWN). From our point of view, these findings might be an impediment for adequate diffusion of the drug into the inner ear. Therefore, the main aim of this study was to evaluate temporal bone anatomy revealed by CBCT in our patients treated with IT steroids and further to possibly find correlations to clinical outcome.

## Materials and methods

### Study design

We retrospectively reviewed all medical records of patients with ISSNHL, who were treated with IT triamcinolone at our institution in 2018. These patients either had experienced prior systemic steroid treatment with poor or absent response (group A; recovery of 10 dB or less of the affected ear four tone average) or were not treated due to contraindications for systemic steroids (group B).

An electronic database search was performed in the hospital information system. Patients’ medical history, pre- and post-therapeutic audiologic examinations and pre-therapeutic temporal bone CBCTs were analyzed. Our aim was to evaluate the individual’s temporal bone anatomy and to possibly find any correlations to the clinical benefit of IT triamcinolone in the primary and secondary treatment of ISSNHL.

Owing to the retrospective nature of the study, patient treatment was already completed at the time of data acquisition. We obtained the approval of our Ethic’s Committee for this retrospective study (No. 8292_BO_K_2019; Medical University Hannover, Germany).

### Demographic data and audiological criteria

We included 31 adult patients, in which 89 IT treatments were performed. Two patients (6%) had only one injection as they did not return for further treatments. The male to female-ratio was 1.2 to 1 and the age averaged 57 years (17–84 years). Twenty-four patients (77%) were assigned group A (prior steroid treatment) and seven patients (23%) were group B-patients (no prior treatment).

Diagnostic criteria for ISSNHL were an abrupt onset of hearing loss of various degrees. We scrutinized reports and information on the prior hearing status, compared the audiogram of the affected ear to the contralateral side and also compared current audiograms to older ones, if available. Patient information on hearing and audiometric inner ear threshold decrease of at least 10 dB in several frequencies was sufficient for putting the diagnosis of ISSNHL. MRI of the cerebellopontine angle and ABR measurements were usually performed in the later course. Four-tone average (FTA) was calculated as an average of the thresholds measured at 0.5, 1, 2, and 3 kHz in PTA before treatment and at the follow-up visit in our outpatient department.

Patients with other etiologies of hearing loss, e.g. autoimmune disorders, vestibular schwannoma or neurologic diseases were excluded.

### Properties of triamcinolone

Triamcinolone acetonid (Triamhexal, 40 mg/ml, Salutas Pharma, Barleben, Germany) is a synthetic glucocorticoid with a pronounced anti-inflammatory activity. The potency on a milligram by milligram comparison is approximately five times that of hydrocortisone. The product is a microcrystalline water suspension which from our point of view results in a better and prolonged adherence to surfaces like the middle ear structures. It is almost insoluble in water and dissolution is known to be slow.

### Technique of IT triamcinolone application

After confirming an intact tympanic membrane and an unattracted middle ear by otoscopy, surface anaesthesia of the ear drum was done placing a medical swab moistened with xylocaine 10% pump spray (Aspen Pharma Trading Ltd., Dublin, Ireland) into the external ear canal with close contact to the tympanic membrane for approximately 10 min. Then, in a hanging down supine position, the head was slightly tilted to the healthy side. Using a 70 mm long 20-Gauge canula (Sterican, Braun Melsungen AG, Melsungen, Germany) and a 1 ml-tuberculin syringe (Luer Duo, Braun Melsungen AG, Melsungen, Germany) the antero-inferior quadrant of the tympanic membrane was pierced and up to 0.5 ml of undiluted triamcinolone solution was applied. Ventilation tubes were not used. During the procedure and afterwards, patients were instructed to avoid swallowing or moving their head out of the hanging down position for approximately 30 min with the aim of keeping the highly concentrated drug in the area of the round and oval window niche. IT triamcinolone injections were repeated once a week for up to 3 consecutive weeks.

### Pre-therapeutic temporal bone CBCT

The routine use of preoperative CBCT was started in the beginning of 2018 (*n* = 27 images, 87% of all 2018 patients).

The pre-therapeutic CBCT imaging was performed in a sitting position on the awake patient using the CBCT (Xoran MiniCAT, Xoran Technologies, USA). It works with a high-performance flat panel detector. The fields of view were 14 × 21 cm. Investigations were performed in a standard mode with a tubal voltage of 125 kVp, 58.8 mAs, a reconstructed slice thickness of 0.3 mm and a rotation of 360° in 20 s. The resulting volume dataset had isotropic voxels of 0.3 mm. Primary reconstructions were done with the device’s application software.

A senior neuro-radiologist (F. G.) evaluated all CBCTs on a visual, descriptive basis without any clinical information on the extent of ISSNHL nor on the post-therapeutic benefit. Results were transferred to a standardized check list which we developed before (Table 1).

**Table 1:** Checklist for the evaluation of the pre-therapeutic cone beam CTs

	Questions		Answers	
1	Evidence on prior ear surgery?		Yes	No
2	Bony overhang at the RWN?		Yes	No
3	Soft tissue inside the RWN?		Yes	No
4	Additional membrane inside the RWN?		Yes	No
5	Soft tissue inside the OWN?		Yes	No
6	Evidence on vestibulocochlear malformations?		Yes	No
	e.g. open semicircular canals, large vestibular aqueducts etc			
7	Evidence on vestibulocochlear ossification?		Yes	No
		*Very good*	*Good*	*Insufficient*
8	Status of middle ear ventilation	completely aerated	Some fluid or soft tissue	No aeration
9	Status of mastoid ventilation	Completely aerated	Partially filled mastoid cells	Sclerotic or no aeration
10	Patency of the Eustachian tube	Full length air column	Half length air column	Less than half length

*RWN* round window niche, *OWN* oval window niche

## Results

### Analysis of pure tone audiometry

**Fig. 1:**
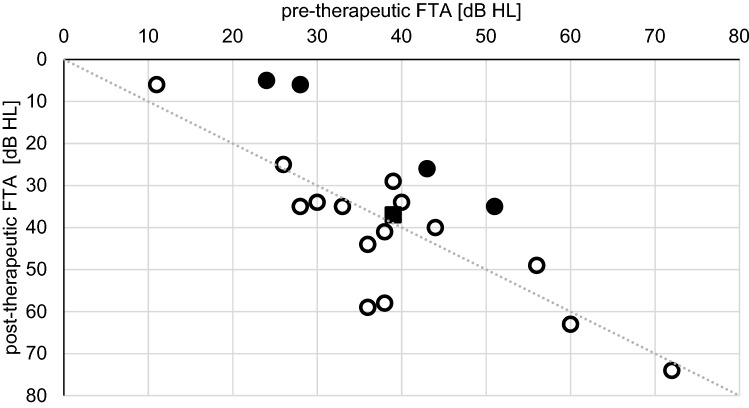
Pre- and post-therapeutic FTA (four-tone average at 0.5, 1, 2 and 3 kHz) of group A-patients. Full circles indicate the patients with a post-therapeutic hearing improvement of more than 15 dB. The black square shows the overall mean of measurements. Only patients with pre- and post-therapeutic audiograms are shown (*n* = 19)

Group A-patients (*n* = 24, 77%) had prior systemic steroid treatment at our (*n* = 7, 29%) or at other departments (*n* = 17, 71%). Due to the insufficient benefit, IT treatment was started on an average 30 days (1–123 days, median 27 days) after the primary event. Pre-therapeutic FTA averaged 39 dB HL (10–75 dB HL) in all patients and 37 dB HL (5–74 dB HL) in the patients, who later returned for post-therapeutic control PTA (*n* = 19, 79%). Among these, we found patients (21%) with an FTA improvement of 15 dB and more, which has to be accounted as clinically relevant. Two patients’ FTA (11%) declined by more than 15 dB and the other 13 (68%) showed insignificant FTA changes (Fig. 1).

**Fig. 2:**
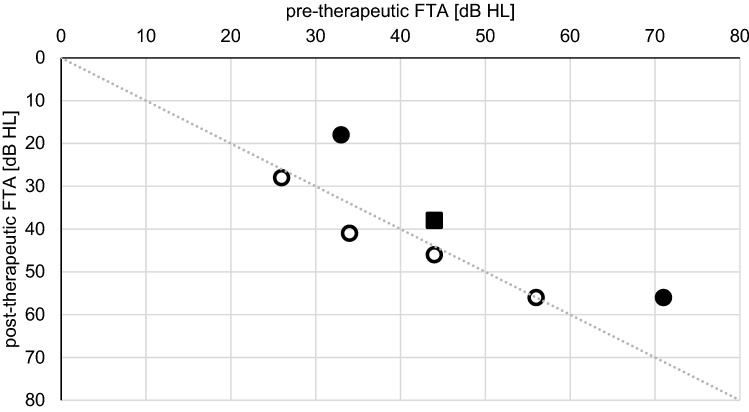
Pre- and post-therapeutic FTA (four-tone average at 0.5, 1, 2 and 3 kHz) of group B-patients. Full circles indicate the patients with a post-therapeutic hearing improvement of more than 15 dB. The black square shows the overall mean of measurements. Only patients with pre- and post-therapeutic audiograms are shown (*n* = 7)

Group B-patients (*n* = 7, 23%) had no prior treatment. IT triamcinolone treatment on average started 16 days (3–45 days, median 14 days) after the primary event. Pre-therapeutic FTA averaged 44 dB HL (26–71 dB HL), post-therapeutic FTA averaged 38 dB HL (18–56 dB HL) in the six patients (85%), which had follow-up. Overall, two patients (33%) showed an FTA improvement of 15 dB. All others (67%) presented no changes in post-therapeutic PTA thresholds (Fig. 2).

On average, 9 days (0–45 days) passed between the decision for the IT treatment and the first injection. Control PTAs (*n* = 25, 81%) were at an average performed 64 days after the last injection (3–256 days).

### Analysis of pre-therapeutic cone beam CT

Overall quality of CBCT images (*n* = 27 images) was rated to be very good (no motion artifacts).

The degree of pneumatization of the temporal bone was rated as ‘very good’ in 17 cases (63%) without any evidence of fluid or soft tissue in the well ventilated bony mastoid cells. ‘Good aeration’ was judged in four cases (15%). Here, some degree of fluid or soft tissue was seen in the well-developed mastoid bone. Six cases (22%) were of ‘insufficient’ aeration as these mastoid air cells were either sclerotic or completely filled with fluid or soft tissue.

Twenty-five patients (93%) showed a ‘very well’ ventilated middle ear without evidence of fluid or soft tissue granulations in the tympanic cavity. Two cases (7%) had insufficient’ middle ear ventilation as these showed larger amounts of fluid or soft tissue.

**Fig. 3:**
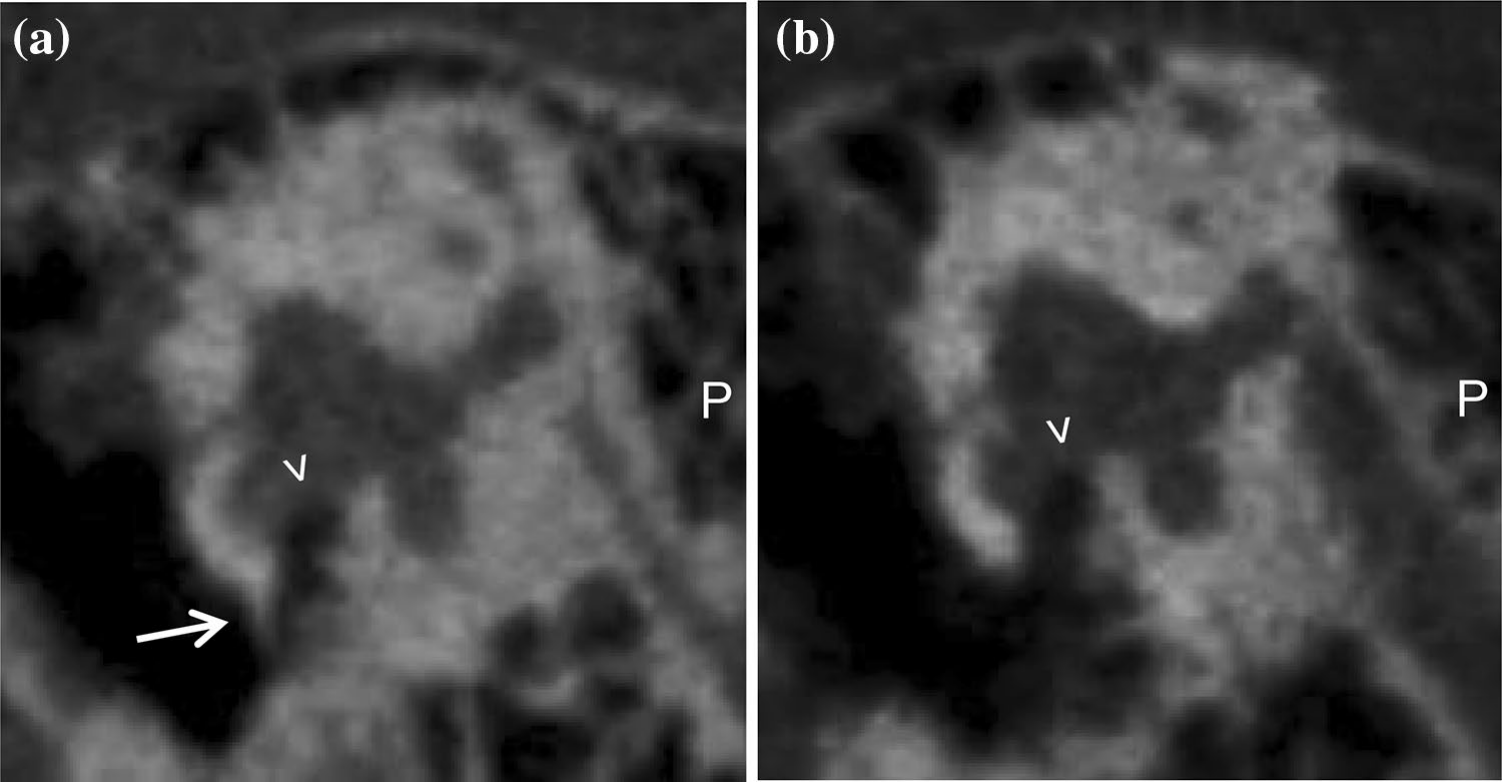
Sagittal slices of temporal bone CBCT. The RWN ( >) can be clearly identified on the right (**a**) and on the left (**b**) patient side. On the right side there is an additional bony overhang ( →), while the left side seems open

**Fig. 4:**
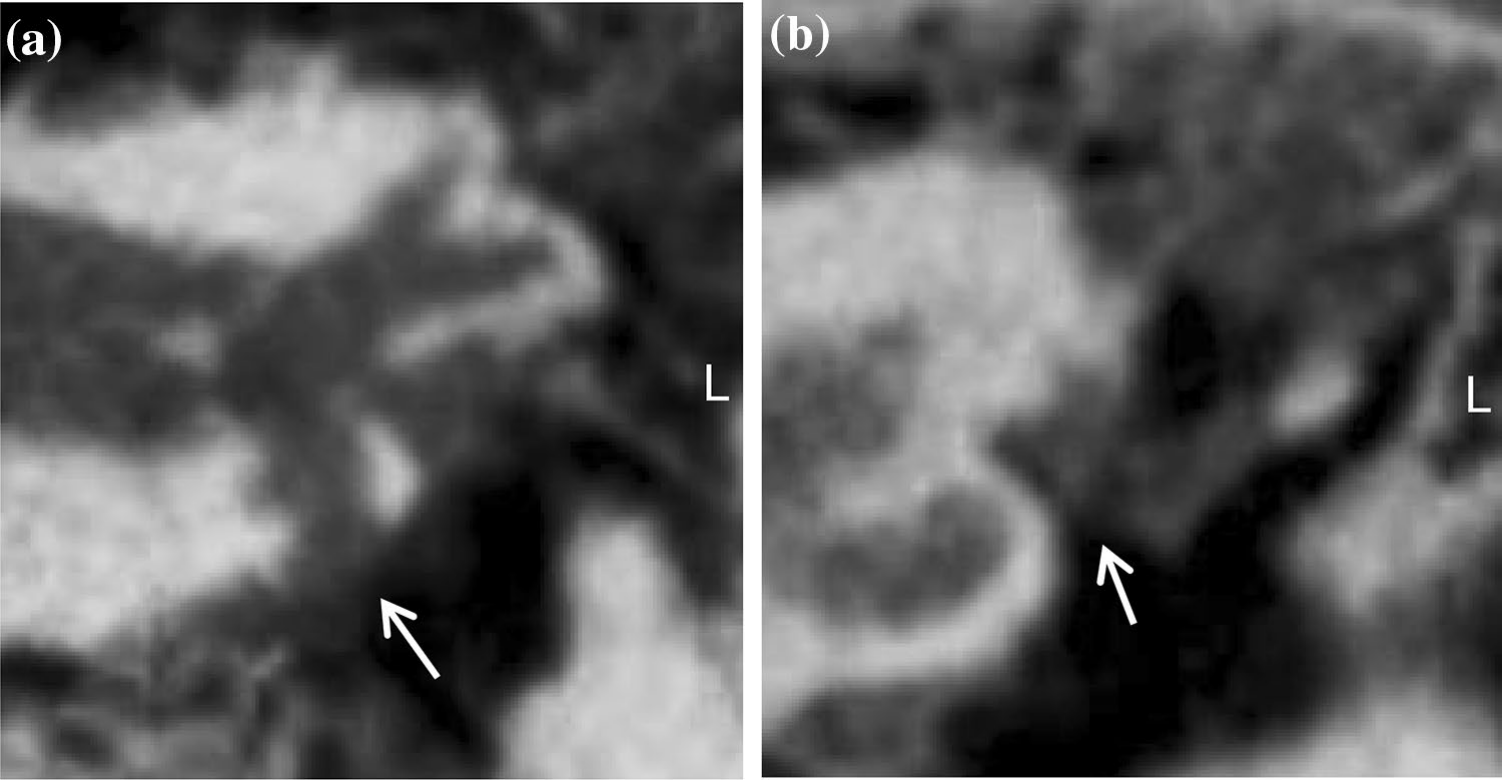
Coronal view of a left temporal bone in CBCT imaging. The RWN (**a**) and the OWN (**b**) are completely opacified ( →). The soft tissue extents to the ossicular chain in (**b**)

**Fig. 5:**
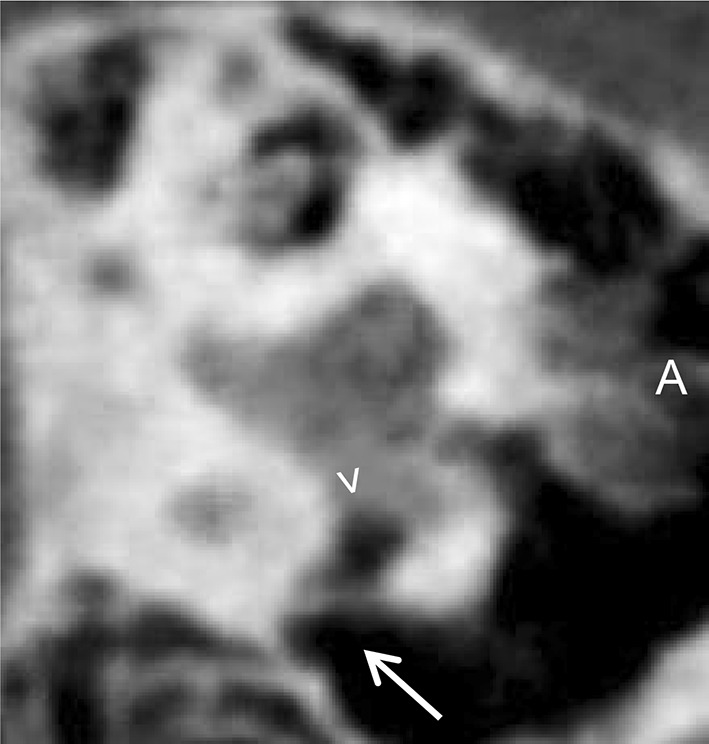
Sagittal view of a left temporal bone in CBCT imaging. The RWN ( >) can be clearly identified but seems capped by a secondary membrane ( →)

Bony overhangs at the RWN were clearly visualized in seven cases (26%, Fig. 3). Granulation tissue inside the RWN, not allowing to identify the round window membrane (RWM), was found in four cases (15%, Fig. 4a). In all other 23 cases (85%) the RWM was clearly visible. Additional soft tissue inside the oval window niche was seen in four cases (15%, Fig. 4b). In one case (4%) it was possible to describe an additional, secondary RWM (Fig. 5). Regarding these 108 radiologically ‘tiny’ items the neuro-radiologist was not able to make a clear decision in nine cases (8%).

The criterion for Eustachian tube patency was a continuous air column from the tympanic cavity to the nasopharynx. This was found in found cases (19%). Ten times (37%) it was possible to trace at least half of the tube. Limited identification of air in less than half of the Eustachian tube length was found in 12 cases (44%).

No vestibulocochlear malformations or pathological ossifications were observed. All semicircular canals were covered by intact bony shells.

One patient (4%) had been operated before on the affected ear. The mastoid was thoroughly drilled out showing a hollow, air-filled cavity. The middle ear also presented ‘very well’ ventilated without granulation tissue. This was a group A-patient with no relevant effect after IT treatment (FTA improvement of 10 dB).

Three of the four group A-patients with a post-therapeutic FTA improvement of 15 dB or more had radiologic imaging. The first patient presented with a bony overhang at the RWN; the second patient with a bony overhang and additional soft tissue at the RWN and an ‘unsufficiently’ aerated mastoid cavity. The third patient was characterized by additional soft tissue at the round and oval window niche and by an opacified tympanomastoid cavity.

All group B-patients with hearing improvement of more than 15 dB did not show pathological findings in the radiologic analysis.

## Discussion

Multiple non-randomized, randomized and even placebo-controlled studies on the benefit of IT glucocorticoids in the primary and secondary treatment of ISSNHL exist [1–9]. Due to the large variances in indication criteria, treatment protocols and the drugs used, no definite recommendations can be drawn and significant hearing improvements can still be questioned. It seems reasonable, that further factors exist, which influence the clinical outcome. From our point of view, anatomic variations like an additional membrane at the RW, an ectatic Eustachian tube, mucosal pathologies of the middle ear or high jugular vein bulbs are relevant as they might limit diffusion processes from the middle into the inner ear.

CBCT offers high accuracy, low radiation and acceptable examination time. In accordance to our experience, several studies confirm equal [21, 22] or even better [23–26] visualization of anatomic landmarks to regular CT scanning, which is facilitated by the ideal 3-D-reconstruction of the complex middle ear structures. Regarding our patient group, all structures we account relevant for IT glucocorticoid treatment were well visualized (Table 1). Image analysis is laborious as tiny structures like bony overhangs, a secondary membrane at the RW or soft tissue at the round or oval window were often visible on a single slice only. This fact might contribute to uncertainty in 8% of all investigated items. The extent of mastoid air cell ventilation was of no importance in our evaluation. Guldner et al. described excellent visualization of very small tympanic structures in middle ears without radiologic pathology or opacity [27]. The limited resolution of our CBCT scans with voxel sizes of 0.3 mm has to be mentioned. More advanced scans have higher resolution, e.g. 0.08 mm voxel size. This allows the identification of even smaller anatomic structures but is not yet established for these indications in our department.

Focusing on the neuro-radiologic analysis we found the evaluated ‘tiny’ anatomical items in all group A-patients who presented with a significant post-therapeutic hearing improvement. Two of the patients had a bony overhang at the RWN, two patients had additional soft tissue at the RWN and one patient also at the OWN. The discrepancy of findings of ‘small pathology’ in the temporal bone and the clinical results in our patients might indicate that anatomical variations at the RWN do not play a significant role in drug diffusion to the inner ear or that other pathways to the inner ear exist. Soft tissue might be an indicator of inflammatory processes, which can be influenced by corticosteroids.

Like other investigators we divided our IT triamcinolone patients into two groups. The first had no benefit from prior systemic steroid treatment (secondary therapy, group A) and the second who presented for primary IT treatment due to contraindications (primary treatment, group B). In comparison to other publications, our subgroups are small as our sampling period was limited to one year [1, 3, 7]. The reason is, that we had been routinely performing CBCT scanning of the temporal bone before IT triamcinolone application since 2018.

A post-therapeutic hearing improvement of more than 15 dB was found in 21% of group A-patients and in 33% of group B-patients. The true role of IT triamcinolone in this clinical setting cannot be clarified, as control groups are missing due to the retrospective character of this study.

In former studies, IT triamcinolone was not evaluated in ISSNHL but it was rated to be helpful in the treatment of Ménière’s disease [28]. Due to the small group size, our results do not reach statistical significance. Furthermore, factors like starting time after the onset of ISSNHL and co-morbidities like diabetes mellitus might play a role in the treatment of ISSNHL. Liebau et al. conclude, that solely the degree of initial hearing loss correlates with post-therapeutic improvement while treatment delay does not play a significant role [5, 6]. The benefit of IT triamcinolone in our cohort is in the range of 12 and 100% as reported by others [1, 3, 4, 7]. However, the comparability of studies is limited due to the wide variety of treatment protocols.

Our findings might further indicate that IT steroids play an inferior role in the treatment of ISSNHL especially regarding the fact of spontaneous recovery. To investigate the true value of IT steroid application a randomized prospective study with a placebo control group is planned also using pre-therapeutic CBCT scanning with even higher resolution.

## Conclusion

Pre-therapeutic imaging is indicated before middle ear interventions like IT steroid treatment for ISSNHL. Radiologic findings like bony overhangs or secondary membranes at the RWN and soft tissue at the round or oval window should be documented, as these might be reasons for inadequate middle to inner ear drug diffusion. However, IT steroid treatment might be beneficial to patients with acute ISSNHL. Gross pathologies at the RWN seem not to have a significant influence on treatment results.
